# Prior ischemic strokes are non-inferior for predicting future ischemic strokes than CHA_2_DS_2_-VASc score in hemodialysis patients with non-valvular atrial fibrillation

**DOI:** 10.1186/s12882-021-02384-0

**Published:** 2021-05-15

**Authors:** Anat Bel-Ange, Shani Zilberman Itskovich, Liana Avivi, Kobi Stav, Shai Efrati, Ilia Beberashvili

**Affiliations:** 1 Internal Department C, affiliated with the Sackler Faculty of Medicine, Yitzhak Shamir Medical Center, Tel Aviv University, Zerifin, Israel; 2 Nephrology Division, affiliated with the Sackler Faculty of Medicine, Tel Aviv University, Yitzhak Shamir Medical Center, 70300 Zerifin, Israel; 3 Internal Department D, affiliated with the Sackler Faculty of Medicine, Tel Aviv University, Yitzhak Shamir Medical Center, Zerifin, Israel; 4 Urology Department, affiliated with the Sackler Faculty of Medicine, Tel Aviv University, Yitzhak Shamir Medical Center, Zerifin, Israel

**Keywords:** CHA_2_DS_2_-VASc, HAS-BLED, Hemodialysis, Stroke, Bleeding

## Abstract

**Background:**

We tested whether CHA_2_DS_2_-VASc and/or HAS-BLED scores better predict ischemic stroke and major bleeding, respectively, than their individual components in maintenance hemodialysis (MHD) patients with atrial fibrillation (AF).

**Methods:**

A retrospective cohort study of a clinical database containing the medical records of 268 MHD patients with non-valvular AF (167 women, mean age 73.4 ± 10.2 years). During the median follow-up of 21.0 (interquartile range, 5.0–44.0) months, 46 (17.2%) ischemic strokes and 24 (9.0%) major bleeding events were reported.

**Results:**

Although CHA_2_DS_2_-VASc predicted ischemic stroke risk in the study population (adjusted HR 1.74 with 95% CI 1.23–2.46 for each unit of increase in CHA_2_DS_2_-VASc score, and HR of 5.57 with 95% CI 1.88–16.49 for CHA_2_DS_2_-VASc score ≥ 6), prior ischemic strokes/transient ischemic attacks (TIAs) were non-inferior in both univariate and multivariate analyses (adjusted HR 8.65 with 95% CI 2.82–26.49). The ROC AUC was larger for the prior ischemic stroke/TIA than for CHA_2_DS_2_-VASc. Furthermore, the CHA_2_DS_2_-VASc score did not predict future ischemic stroke risks in study participants who did not previously experience ischemic strokes/TIAs (adjusted HR 1.41, 95% CI: 0.84–2.36). The HAS-BLED score and its components did not have predictive abilities in discriminating bleeding risk in the study population.

**Conclusions:**

Previous ischemic strokes are non-inferior for predicting of future ischemic strokes than the complete CHA_2_DS_2_-VASc score in MHD patients. CHA_2_DS_2_VASc scores are less predictive in MHD patients without histories of CVA/TIA. HAS-BLED scores do not predict major bleeding in MHD patients. These findings should redesign approaches to ischemic stroke risk stratification in MHD patients if future large-scale epidemiological studies confirm them.

## Introduction

Atrial fibrillation (AF) is a common problem in end-stage kidney disease (ESKD) patients receiving maintenance hemodialysis (MHD) treatment [1–3] that increases the risk of cerebrovascular accident (CVA) and death [3, 4]. Its prevalence is reported as 11.6% and the overall incidence as 2.7/100 patient-years in this population [1]. The causes of AF in MHD patients include common risk factors for both AF and ESKD (e.g. age, hypertension, diabetes, congestive heart failure (CHF)) as well as risk factors directly related to dialysis (such as changes in the fluid balance, electrolyte imbalance leading to sympathetic nervous system activation, chronic inflammation and structural changes in the heart) and discussed elsewhere [5, 6]. Compared to the general population, MHD patients are at increased risk of bleeding due to platelet dysfunction, coagulation factor levels and associated gastrointestinal diseases [7]. Therefore, the provision of chronic anticoagulation drugs to prevent ischemic stroke, which is recommended in a general population with AF, is not always recommended in MHD patients [8–10].

The CHA_2_DS_2_-VASc and HAS-BLED scores allow for quickly evaluating the risks versus benefits of administrating anticoagulants in the general population. The CHA_2_DS_2_-VASc score is the improved version of CHADS_2_ and includes clinical predictors for assessing the risk of thromboembolic stroke in patients with non-rheumatic AF [11] and has been verified in a number of large epidemiological studies [12, 13]. The HAS-BLED score was also developed and validated in the general population to assess the annual risk of significant bleeding in patients with atrial fibrillation [14]. However, only a few studies evaluated the aforementioned scoring systems in ESKD patients receiving MHD treatment [15–17].

Since ESKD requiring MHD carries up to a tenfold greater risk of stroke than normal renal function [18], the CHA_2_DS_2_-VASc score loses its advantage over the CHADS_2_ score to identify dialysis patients who are at low risk for ischemic stroke. It will only reveal dialysis patients with high risk of ischemic stroke. The HAS-BLED score also appears to have apoor predictive ability for hemorrhage risk discrimination in MHD population [17]. One main obstacle in using the CHA_2_DS_2_-VASc and/or HAS-BLED scores in the ESKD population is that the risk factor weighting of the individual components within these scoring systems in MHD patients is not the same as in the general population where these scores were developed. Therefore, the performance of these scores might not improve and may even lower the predictive performance of their components in MHD patients. To address this concern, we aimed to test in MHD patients whether CHA_2_DS_2_-VASc and/or HAS-BLED scores better predict ischemic strokes and major bleeding, respectively, than their individual components.

## Methods

### Study design and patients

We performed a historical prospective cohort study of ESKD patients receiving MHD treatment from January 2005 to January 2019 in a single dialysis center. This study was approved by our local institutional ethics committee. We were exempt from written informed consents due to the study’s retrospective design.

All methods were carried out in accordance with relevant guidelines and regulations.

**Fig. 1:**
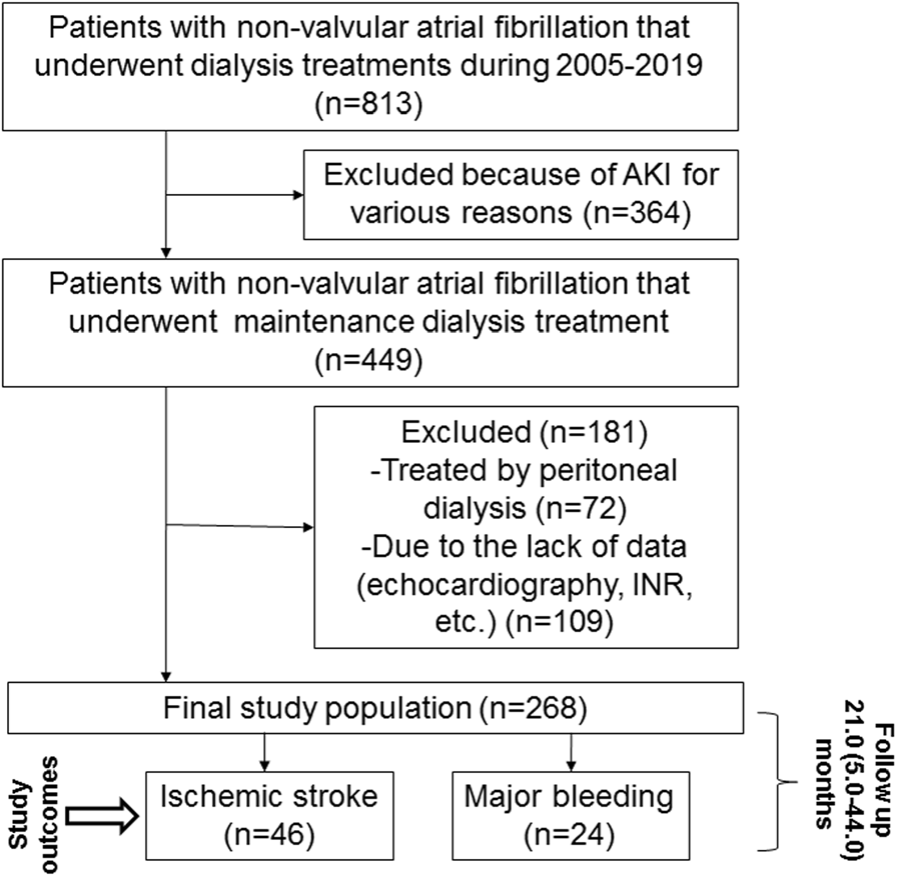
Flow diagram of the study

The information included patient demographics, medications, clinical laboratory values, echocardiography data, dialysis treatment records, and all comorbidities available to the dialysis unit. Patients included in the study were those > >18 years old with a documented history of non-valvular AF who received thrice-weekly in–center MHD for > 2 months. Patients with chronic coagulation problems and/or with mitral stenosis or mechanical valves who needed chronic anticoagulation therapy were excluded from the study. The first (baseline) visit for each patient was the calendar date on which the patient’s dialysis vintage was 60 days. Follow-up time began on the date of entry into the cohort. Patients were followed until an ischemic stroke, a bleeding event, time of death or censored due to kidney transplantation, loss of follow-up, or until December 2019. From 813 patients with non-valvular AF receiving dialysis during the cohort period, 545 were non-eligible (364 patients with AKI for various reasons, 72 patients treated with PD and 109 patients lacking data, especially an echocardiography). A flow chart of the study is presented in Fig. 1**.** A total of 268 MHD patients (167 women) with a mean age of 73.4 ± 10.2 years were included in the statistical analysis. These patients contributed a total of 489 patient-years of at-risk time, and the median follow-up was 21.0 (interquartile range, 5.0–44.0) months. During the follow-up, 46 (17.2%) ischemic strokes and 24 (9.0%) major bleeding events were reported.

### CHA_2_DS_2_-VASc and HAS-BLED scores

The CHA_2_DS_2_-VASc score was the sum of points after adding one point each for heart failure, hypertension, diabetes, vascular disease, age 65–74 years, and female sex, and two points each for previous thromboembolism and age ≥ 75 years. This score thus ranged from 0 to 9 [11].

The HAS-BLED score incorporates risk factors for bleeding (hypertension, abnormal renal/liver function, stroke, bleeding history or predisposition, labile international normalized ratio, elderly (> 65 years), and drugs/alcohol concomitantly). We calculated the score as the sum of points after assigning one point to each of the score’s aforementioned components, as originally recommended [14]. This score also ranges from 0 to 9.

### Outcomes and comorbidity index

Diagnosis of ischemic stroke and/or transient ischemic attack (TIA) required consultation of a neurologist. A new stroke required radiological documentation by brain imaging studies, including computed tomography or magnetic resonance imaging.

Bleeding outcome was defined as any kind of bleeding (such as intracerebral bleeding, gastrointestinal bleeding, retroperitoneal bleeding etc.) requiring hospitalization, or fatal bleeding.

We determined the comorbidity index, which was recently developed by Liu et al. [19] and validated specifically for dialysis patient populations, as a measure of comorbid conditions.

### Laboratory evaluation

Blood samples were obtained from non-fasting patients on a midweek day predialysis, with the exception of postdialysis serum urea nitrogen to calculate urea kinetics. All biochemical analyses were measured by an automatic analyzer.

### Statistical analyses

Data are expressed as means ± SDs for normally distributed data, medians and interquartile ranges (quartiles 1–3) for variables that did not follow a normal distribution, or frequencies for categorical variables.

Normally distributed continuous variables were compared between the two groups using a two–sided t test. Non-normally distributed continuous variables were compared between the two groups by nonparametric Mann–Whitney U tests. Chi-squared tests were used for comparison of categorical variables.

A receiver operating characteristic (ROC) curve was generated for the most accurate discrimination of ischemic stroke/major bleeding risk for the scores and its components separately. The area under the ROC curve (AUC) indicated the probability of discriminating a risk. The cutoffs for the most accurate discrimination of ischemic stroke/major bleeding risk were derived from these ROC AUCs. These cutoff points were used to calculate the sensitivity and specificity of each score (CHA_2_DS_2_-VASc and HAS-BLED) in predicting ischemic stroke/major bleeding. To determine the posttest probability, the positive likelihood ratio (LR+) and negative likelihood ratio (LR-) were calculated as follows [20]:

LR+ = sensitivity/(1–specificity) and

LR- = (1 – sensitivity)/specificity.

To adapt the CHA_2_DS_2_-VASc and HAS-BLED scores for predicting outcome variable (ischemic stroke or major bleeding, respectively) in Kaplan-Meier estimator, we have divided them into tertiles in low-, intermediate- and high-risk groups by an increase of 3 points. Kaplan–Meier curves for the risk groups were computed for ischemic stroke and bleeding within 12 years of follow-up as outcome. Univariate and multivariate Cox regression analyses were used to provide univariate, adjusted hazard ratios (HR) and 95% confidence intervals (CI) for each independent risk factor. The confounders for multivariable models were chosen from the univariate data analyses sets with *p* < 0.25 and/or based on a priori knowledge of the relationship with the outcome measure.

All statistical tests were two-sided, with a value of *p* < 0.05 defining significance.

All statistical analyses were performed using SPSS software, version 18.0 (IBM SPSS, Chicago, IL).

## Results

Of the 268 MHD patients in this study, 61.0% were female and 68.4% had diabetes. The participants had a mean CHA_2_DS_2_-VASc score of 5.1 ± 1.7 and a mean HAS-BLED score of 4.5 ± 1.1 (Tables 1 and 2, respectively). The patients with ischemic stroke had higher Kt/V and CHA_2_DS_2_-VASc scores compared to the patients without ischemic strokes, with no statistically significant differences in demographics, comorbidities, functional status, time since AF diagnosis, antiplatelets use, hemoglobin and platelets levels between the groups with and without ischemic strokes (Table 1). The patients with major bleeding events had lower Kt/V and higher functional activity compared to patients without major bleeding events, with no statistically significant differences in demographics, comorbidities, time since AF diagnosis, warfarin, enoxaparin and/or antiplatelets use, INR levels and HAS-BLED scores between the groups with and without major bleeding events (Table 2).

**Table 1:** Demographic and clinical characteristics of the study population with AF at baseline according to ischemic stroke event

Variables	Without Stroke (*n* = 222)	With Stroke (*n* = 46)	*P* value
Age (y)	73.4 ± 10.5	73.2 ± 8.8	0.89
Gender male n (%)	86 (39)	19 (41)	0.74
DM n (%)	150 (68)	35 (76)	0.30
Dialysis vintage (years)	0.0 (0.0–3.0)	0.0 (0.0–2.0)	0.66
Kt/V	1.34 ± 0.18	1.41 ± 0.18	0.02
Vascular access			0.70
Arterio-venous fistula n (%)	78 (35)	21 (46)	
Arterio-venous graft n (%)	16 (7)	4 (9)	
Central venous Catheter n (%)	128 (58)	21 (46)	
Heparin dose during HD session (units)	2500 (1250–2500)	2500 (0–2500)	0.28
Comorbidity index	8.7 ± 3.3	9.1 ± 2.9	0.44
Functional status (need in nursing care) n (%)	120 (55)	28 (61)	0.41
Dementia n (%)	36 (16)	10 (22)	0.39
A-V access thrombosis n (%)	42 (19)	15 (33)	0.06
Left atrium size (cm)	4.3 ± 0.7	4.4 ± 0.5	0.94
Time since AF diagnosis (years)	3.0 (1.0–7.0)	4.0 (1.0–7.8)	0.45
All-cause death n (%)	175 (79)	41 (89)	0.15
Warfarin use n (%)	76 (34)	15 (33)	0.87
Aspirin use n (%)	126 (57)	27 (59)	0.86
Clopidogrel use n (%)	45 (20)	15 (33)	0.29
Dual antiplatelet therapy n (%)	33 (15)	7 (15)	0.96
Enoxaparin use n (%)	29 (13)	6 (13)	0.97
INR	1.2 (1.1–1.5)	1.2 (1.1–1.5)	0.95
Hb (g/dL)	10.0 ± 1.8	10.6 ± 1.5	0.12
Platelets(× 10^3^/mm^3^)	191.1 ± 70.3	216.4 ± 80.1	0.27
Mean CHA_2_DS_2_-VASc score	4.9 ± 1.6	5.7 ± 1.6	0.005
CHA_2_DS_2_-VASc score			0.06
0 n (%)	1 (0.5)	0 (0)	
1 n (%)	5 (2.3)	0 (0)	
2 n (%)	11 (4.9)	1 (2.2)	
3 n (%)	20 (9.0)	4 (8.7)	
4 n (%)	44 (19.8)	6 (13.0)	
5 n (%)	64 (28.8)	7 (15.2)	
6 n (%)	40 (18.0)	16 (34.8)	
7 n (%)	25 (11.3)	5 (10.9)	
8 n (%)	10 (4.5)	5 (10.9)	
9 n (%)	2 (0.9)	2 (4.3)	

Continuous variables with normal distribution are expressed as means (SDs), as medians (interquartile ranges) for non–normally distributed data, and categorical variables are expressed as percentages

*Abbreviations*: *AF* atrial fibrillation, *HD* hemodialysis, *DM* diabetes mellitus, *GI* gastrointestinal, *PPI* proton pump inhibitor, *Hb* hemoglobin

**Table 2:** Demographic and clinical characteristics of the study population with AF at baseline according to major bleeding event

Variables	Without bleeding (*n* = 244)	With Bleeding (*n* = 24)	*P* value
Age (y)	7 3.7 ± 10.0	71.9 ± 12.0	0.42
Gender male n (%)	94 (39)	10 (42)	0.83
DM n (%)	169 (70)	16 (67)	0.82
Dialysis vintage (years)	0.0 (0.0–3.0)	0.0 (0.0–3.8)	0.83
Kt/V	1.36 ± 0.17	1.26 ± 0.25	0.006
Vascular access			0.46
Arterio-venous fistula n (%)	89 (37)	11 (46)	
Arterio-venous graft n (%)	17 (7)	4 (17)	
Central venous catheter n (%)	136 (56)	9 (37)	
Heparin dose during HD session (units)	2500 (0–2500)	1250 (0–2500)	0.33
Comorbidity index	8.7 ± 3.2	9.0 ± 3.1	0.63
Functional status (need in nursing care) n (%)	142 (59)	9 (37)	0.03
Dementia n (%)	43 (18)	4 (17)	0.90
A-V access thrombosis n (%)	50 (21)	7 (29)	0.43
Left atrium size (cm)	4.3 ± 0.6	4.4 ± 0.5	0.54
Time since AF diagnosis (years)	4.0 (1.0–7.0)	2.0 (0.0–6.0)	0.26
All-cause death n (%)	193 (80)	21 (88)	0.59
Warfarin use n (%)	82 (34)	9 (37)	0.82
Aspirin use n (%)	137 (57)	16 (67)	0.39
Clopidogrel use n (%)	54 (22)	7 (29)	0.45
Dual antiplatelet therapy n (%)	34 (14)	7 (29)	0.07
Enoxaparin use n (%)	31 (13)	3 (12)	0.69
PPI n (%)	130 (54)	17 (71)	0.13
INR	1.2 (1.1–1.4)	1.3 (1.1–1.7)	0.41
Hb (g/dL)	10.1 ± 1.7	9.5 ± 1.7	0.40
Platelets(×10^3^/mm^3^)	196.8 ± 73.4	184.9 ± 63.6	0.47
Mean HAS-BLED score	4.5 ± 1.2	4.4 ± 1.1	0.54
HAS-BLED score			0.92
0 n (%)	0 (0)	0 (0)	
1 n (%)	2 (0.8)	0 (0)	
2 n (%)	3 (1.2)	1 (4.2)	
3 n (%)	41 (16.5)	4 (16.7)	
4 n (%)	76 (31.0)	8 (33.3)	
5 n (%)	76 (31.0)	7 (29.4)	
6 n (%)	36 (15.4)	4 (16.7)	
7 n (%)	9 (3.7)	0 (0)	
8 n (%)	1 (0.4)	0 (0)	
9 n (%)	0 (0)	0 (0)	

Continuous variables with normal distribution are expressed as means (SDs), as medians (interquartile ranges) for non–normally distributed data, and categorical variables are expressed as percentages

*Abbreviations*: *AF* atrial fibrillation, *HD* hemodialysis, *DM* diabetes mellitus, *GI* gastrointestinal, *PPI* proton pump inhibitor, *Hb* hemoglobin

We initially compared the discriminative ability of CHA_2_DS_2_-VASc and HAS-BLED scores and their own components in predicting the risk of future ischemic strokes and major bleeding events, respectively (Table 3). The ROC AUC was larger for the prior ischemic stroke/TIA than for the CHA_2_DS_2_-VASc. Furthermore, CHA_2_DS_2_-VASc scores above 6 (the cutoff for the most accurate discrimination of ischemic stroke risk derived from ROC AUC) showed a specificity of less than 70% whereas prior ischemic stroke/TIA expressed at least a similar specificity (73%) in predicting future ischemic strokes. The posttest probability of ischemic strokes in patients at risk according to the CHA_2_DS_2_-VASc and having prior ischemic stroke/TIA was examined by the likelihood ratios. Prior ischemic stroke/TIA had a higher positive likelihood ratio (=2.25) and a lower negative likelihood ratio (=0.53) than CHA_2_DS_2_-VASc scores. This means those future ischemic strokes are 2.25 times more likely to develop in MHD patients with prior ischemic stroke/TIA than in MHD patients without a history of an ischemic stroke/TIA. At the same time, MHD patients without prior ischemic strokes/TIAs had a twofold decrease in the odds of having future ischemic stroke than MHD patients with ischemic strokes/TIAs in the past. Neither HAS-BLED nor its components performed as predictors of future major bleeding in our study population according to the ROC AUCs (Table 3). These results did not change significantly for the CHA_2_DS_2_-VASc and HAS-BLED scores after separate analysis of a group of patients who are not on chronic anticoagulation (including warfarin) (Table 3).

**Table 3:** Comparing the AUC of the CHA_2_DS_2_-VASc and HAS-BLED scores with their components in predicting ischemic stroke risk or major bleeding respectively, in the study population

Variable	All patients (*n* = 268)			Without warfarin (*n* = 180)		
	AUC	95% CI	*P* value	AUC	95% CI	*P* value
**CHA**_**2**_**DS**_**2**_**-VASc components**						
CHF (yes)	0.49	0.40–0.58	0.84	0.46	0.35–0.58	0.52
Hypertension (Yes)	0.50	0.41–0.60	0.95	0.49	0.38–0.60	0.84
Age ≥ 75 years (yes)	0.49	0.40–0.58	0.78	0.47	0.36–0.58	0.62
DM (yes)	0.54	0.45–0.63	0.36	0.52	0.41–0.64	0.68
Prior stroke or TIA (yes)	0.67	0.58–0.76	< 0.001	0.67	0.56–0.78	0.003
Vascular disease (yes)	0.50	0.40–0.59	0.92	0.49	0.38–0.61	0.91
Age 65–74 years (yes)	0.52	0.43–0.61	0.66	0.51	0.40–0.63	0.81
Sex (female)	0.52	0.42–0.61	0.71	0.48	0.37–0.59	0.73
***CHA***_***2***_***DS***_***2***_***-VASc score***	0.63	0.54–0.72	0.006	0.59	0.48–0.70	0.11
**HAS-BLED components**						
Hypertension (yes)	0.46	0.33–0.59	0.50	0.45	0.29–0.61	0.54
Abnormal renal function (yes)	0.50	0.35–0.65	1.00	0.50	0.35–0.65	1.00
Abnormal liver function (yes)	0.51	0.38–0.63	0.92	0.51	0.36–0.67	0.85
Prior stroke (yes)	0.44	0.32–0.55	0.31	0.43	0.29–0.57	0.37
Prior major bleeding (yes)	0.59	0.47–0.72	0.15	0.60	0.45–0.76	0.19
Labile INR (yes)	0.49	0.38–0.62	0.95	0.50	0.34–0.65	0.96
Age > 65 years (yes)	0.40	0.27–0.52	0.09	0.47	0.31–0.63	0.71
Prior alcohol or drug usage (yes)	0.53	0.40–0.65	0.68	0.52	0.36–0.68	0.81
Medication usage predisposing to bleeding (yes)	0.46	0.31–0.52	0.55	0.54	0.39–0.69	0.58
***HAS-BLED score***	0.47	0.35–0.57	0.62	0.49	0.34–0.65	0.94

CHA_2_DS_2_-VASc≥6 had a sensitivity of 61% and a specificity of 65% for predicting ischemic stroke, with +LR 1.75 and –LR 0.60

Prior stroke or TIA had a sensitivity of 61% and a specificity of 73% for predicting ischemic stroke, with +LR 2.25 and –LR 0.53

HAS-BLED≥4 had a sensitivity of 54% and a specificity of 50% for predicting major bleeding, with +LR 1.09 and –LR 0.91

Prior major bleeding had a sensitivity of 42% and a specificity of 76% for predicting major bleeding, with +LR 1.76 and –LR 0.76

*Abbreviations*: *CHF* congestive heart failure, *DM* diabetes mellitus, *TIA* transient ischemic attack

**Fig. 2:**
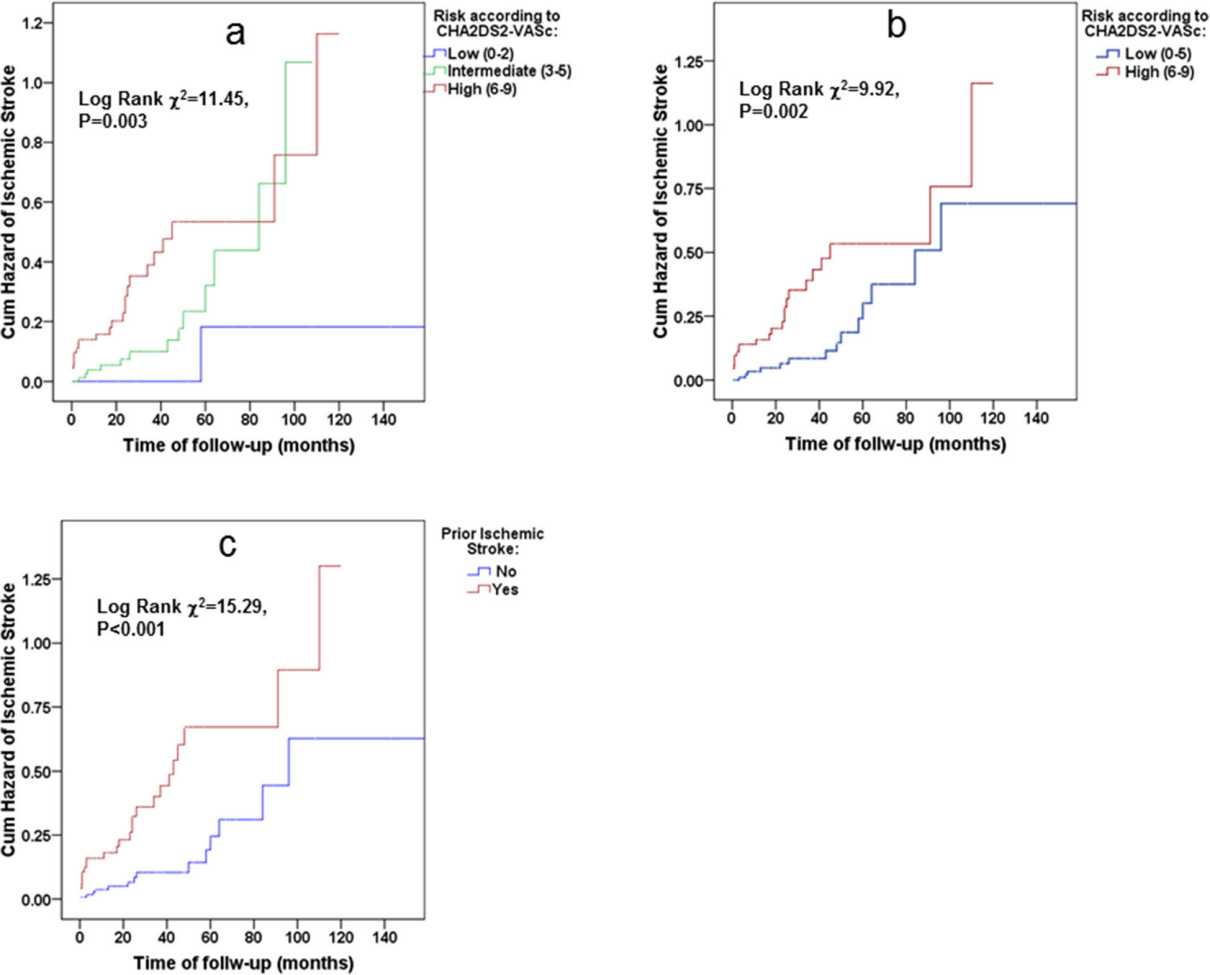
Kaplan–Meier curves for cumulative hazard of ischemic stroke in the study population (*n* = 268, event *n* = 46) according to CHA_2_DS_2_VASc-score stratified as low-, intermediate- and high-risk groups (**a**), as low and high-risk groups according to the cut-off value obtained from AUC ROC analysis (**b**) and according to the history of prior ischemic stroke (**c**)

**Fig. 3:**
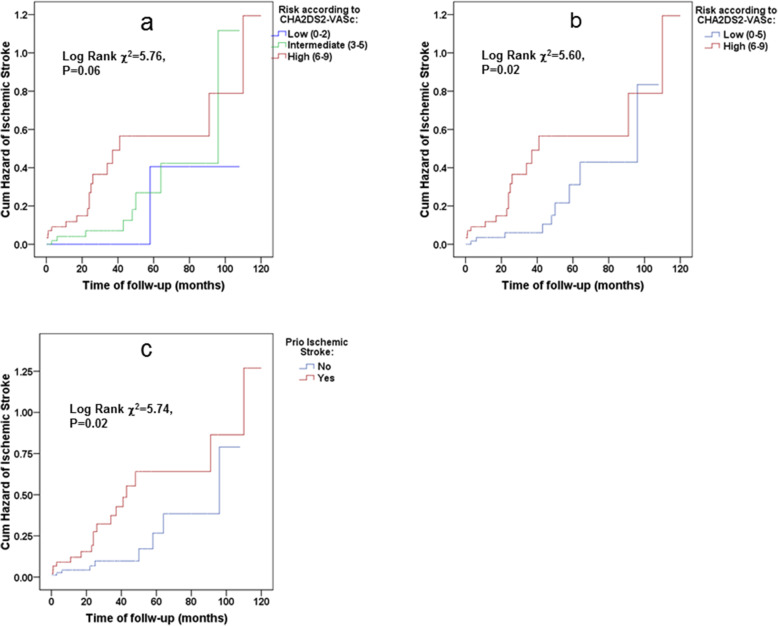
Kaplan–Meier curves for cumulative hazard of ischemic stroke in the study population without chronic anticoagulation (*n* = 176, event *n* = 31) according to CHA_2_DS_2_VASc-score stratified as low-, intermediate- and high-risk groups (**a**), as low and high-risk groups according to the cut-off value obtained from AUC ROC analysis (**b**) and according to the history of prior ischemic stroke (**c**)

**Fig. 4:**
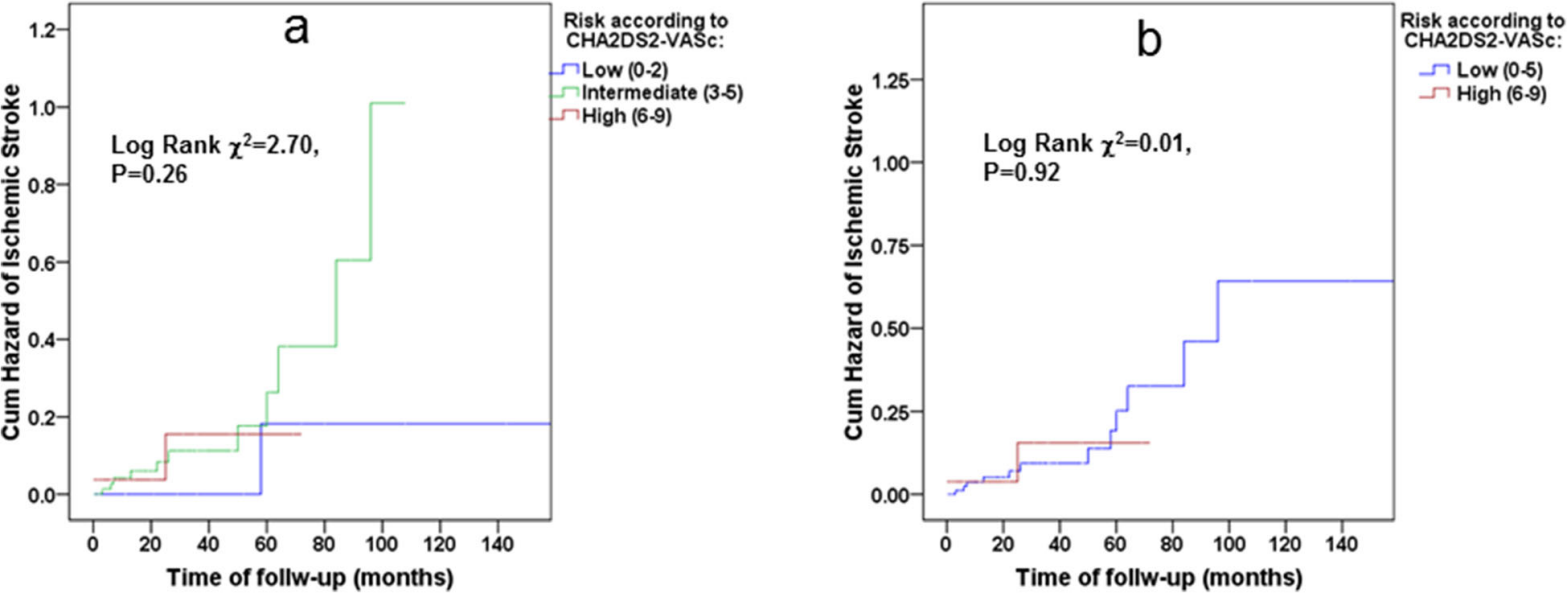
Kaplan–Meier curves for cumulative hazard of ischemic stroke in the study population without past history of ischemic stroke (*n* = 181, event *n* = 18) according to CHA_2_DS_2_VASc-score stratified as low-, intermediate- and high-risk groups (**a**) and as low and high-risk groups according to the cut-off value obtained from AUC ROC analysis (**b**)

Figure 2 illustrates the cumulative hazards of ischemic stroke according to CHA_2_DS_2_VASc score stratified as low-, intermediate- and high-risk groups per increase of 3 points in the score (a), dichotomized as low and high-risk groups according to the cut-off value obtained from AUC ROC analysis (b) and prior ischemic stroke (c), indicating an increasing CHA2DS2-VASc score as well as prior ischemic stroke as associated with increased risk for ischemic stroke. Similar results were demonstrated in a study population without chronic anticoagulation (Fig. 3a, b and c, respectively). However, the CHA2DS2-VASc score did not predict risks of future ischemic strokes in study participants who had not previously experienced an ischemic stroke/TIA (adjusted HR 1.41, 95% CI: 0.84–2.36, see also Fig. 4a and b). And this despite the mean ± 2SD of the CHA2DS2-VASc score that ranged in these patients from 2 to 7, which is recognized as high-risk in the general population. Table 4 lists the hazard ratios and 95% CIs of the CHA_2_DS_2_-VASc scores as continuous variables, as well as after dichotomization, using the cutoff obtained by the ROC analysis. It also lists prior ischemic strokes/TIAs for predicting future ischemic strokes in the study population using univariate and multivariable Cox proportional hazards models. Although the CHA_2_DS_2_-VASc score was a valid score in predicting the risk of ischemic stroke in the study population (adjusted HR 1.74 with 95% CI 1.23 to 2.46, *p* = 0.002 for each unit of increase in the CHA_2_DS_2_-VASc score, and a HR of 5.57 with 95% CI 1.88 to 16.49, *p* = 0.002 for CHA_2_DS_2_-VASc score ≥ 6), prior ischemic strokes/TIAs were found to be no less informative predictors in both univariate and multivariate analyses (adjusted HR 8.65 with 95% CI 2.82 to 26.49, *p* < 0.001). These associations did not lose significance even after all-cause death was inserted as a competing risk in the Cox models (model 3). Note that these results were identical in a study population without chronic anticoagulation (Table 4).

**Table 4:** Comparison of the CHA_2_DS_2_-VASc score and prior stroke and/or TIA in predicting of ischemic stroke according to multivariable Cox proportional hazard models in the study population

Variable	All patients (*n* = 268)			Without warfarin (*n* = 180)		
	HR	95% CI	*P* value	HR	95% CI	*P* value
**CHA**_**2**_**DS**_**2**_**-VASc score (↑ per unit)**						
*Crude*						
CHA_2_DS_2_-VASc	1.28	1.08–1.53	0.006	1.11	0.90–1.37	0.32
*Multivariable models*						
1. Crude +Hb + Co-morbidity index	1.66	1.22–2.28	0.001	1.32	0.90–1.92	0.16
2. 1 + Time since AF diagnosis + Kt/V + A-V access thrombosis in the past	1.73	1.23–2.45	0.002	1.22	0.81–1.85	0.34
3. 2 + All-cause death	1.74	1.23–2.46	0.002	1.22	0.81–1.84	0.35
**CHA**_**2**_**DS**_**2**_**-VASc score (≥6)**						
*Crude*						
CHA_2_DS_2_-VASc	2.75	1.43–5.31	0.003	2.61	1.14–5.94	0.02
*Multivariable models*						
1. Crude + Hb + Co-morbidity index	5.81	2.04–16.60	0.001	6.03	1.51–24.05	0.01
2. 1 + Time since AF diagnosis + Kt/V + A-V access thrombosis in the past	5.56	1.89–16.36	0.002	4.92	1.09–22.22	0.04
3. 2 + All-cause death	5.57	1.88–16.49	0.002	5.00	1.09–22.88	0.04
**Prior stroke and/or TIA**						
*Crude*						
prior stroke and/or TIA (yes)	3.44	1.78–6.65	< 0.001	2.63	1.56–5.97	0.02
*Multivariable models*						
1. Crude +Hb + Co-morbidity index	8.55	2.93–24.97	< 0.001	8.86	1.95–40.36	0.005
2. 1 + Time since AF diagnosis + Kt/V + A-V access thrombosis in the past	8.64	2.83–26.36	< 0.001	8.21	1.64–40.95	0.01
3. 2 + All-cause death	8.65	2.82–26.49	< 0.001	8.23	1.65–41.16	0.01

*Abbreviations*: *Hb* hemoglobin, *AF* atrial fibrillation, *TIA* transient ischemic attack

**Fig. 5:**
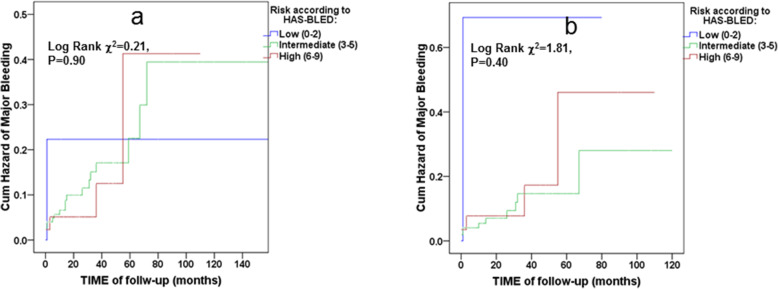
Kaplan–Meier curves for cumulative hazard of major bleeding in the whole study population (*n* = 268, event *n* = 24) (**a**) and in the study population without chronic anticoagulation (*n* = 176, event *n* = 15) (**b**) according to HAS-BLED score stratified as low-, intermediate- and high-risk groups

Kaplan–Meier curves for cumulative hazard of major bleeding in the whole study population (Fig. 5a), as well as in the study population without chronic anticoagulation (Fig. 5b) did not demonstrate any associations between the HAS-BLED score and future major bleeding events. The Cox proportional hazard models of the HAS-BLED score compared to prior major bleeding in predicting future major bleeding (Table 5) in the study population, showed that the HAS-BLED score did not perform as a predictor of major bleeding events. Although predicting future bleeding events by major bleeding in the past was statistically marginal in the univariate analysis, it became meaningless immediately after adjusting to INR levels in the second Cox regression model (Table 5).

**Table 5:** Comparison of the HAS-BLED score and prior major bleeding in predicting of future major bleeding event according to multivariable Cox proportional hazard models in the study population

Variable	HR	95% CI	*P* value
**HAS-BLED score**			
*Crude*			
HAS-BLED (↑ per unit)	0.89	0.63–1.27	0.53
*Multivariable models*			
1. Crude + INR	0.80	0.54–1.19	0.27
2. 1 + Comorbidity index + Functional status	0.70	0.44–1.12	0.19
3. 2 + Dual antiplatelet therapy + Kt/V + PPI use	0.76	0.47–1.20	0.24
4. 3 + All-cause death	0.76	0.48–1.22	0.25
**Prior Major Bleeding**			
*Crude*			
Prior Major Bleeding (yes)	2.30	1.00–5.27	0.05
*Multivariable models*			
1. Crude + INR	1.65	0.62–4.24	0.30
2. 1 + Comorbidity index + Functional status	2.05	0.63–6.72	0.23
3. 2 + Dual antiplatelet therapy + Kt/V + PPI use	2.10	0.67–6.59	0.20
4. 3 + All-cause death	2.07	0.62–6.92	0.24

*Abbreviations*: *INR* international normalized ratio, *PPI* proton-pump inhibitor

## Discussion

The study’s major finding is that prior ischemic strokes predict future ischemic strokes no less good than the complete CHA_2_DS_2_-VASc score in MHD patients. This may redesign the approach to ischemic stroke risk stratification in MHD patients if future large-scale epidemiological studies confirm these findings. HAS-BLED and its components failed to predict major bleeding in our population. This study extends our risk assessment knowledge, since the individual components of the CHA2DS2-VASc and HAS-BLED scores were yet to be compared to full scores in the MHD population.

Our study agrees with a study showing that CHA_2_DS_2_-VASc scores predict ischemic strokes in Taiwanese MHD patients with AF [15]. The CHA_2_DS_2_-VASc levels, ischemic stroke rates and also ROC AUC for CHA_2_DS_2_-VASc in discriminating ischemic stroke risk are similar in both studies. However, individual CHA_2_DS_2_-VASc components were not examined in the Taiwanese study.

Picciniet al. developed the new R_2_CHADS_2_ scoring system in patients with nonvalvular AF and chronic kidney disease (with eGFRs above 30 ml/min/1.73m^2^) participating in the ROCKET study [21]. Comparing clinical factors associated with stroke and/or systemic embolism, the authors reported previous strokes or TIAs as the strongest and independent predictor (with HR of 1.825 with 95% CI of 1.514 to 2.199) that even overcame renal dysfunction and CHADS_2_ as a whole score [21]. The R_2_CHADS_2_ score was further examined in patients with advanced chronic kidney disease (CKD) (eGFR below 30 ml/min/1.73m^2^) [22]. Also in this study, a history of previous stroke almost tripled the risk for subsequent strokes, TIAs or other central thrombosis (risk ratio reported as 2.9, with 95% CI of 2.26 to 3.71) and emerged as a stronger risk factor than advanced CKD (adjusted risk ratio for CKD reported as 1.30, with 95% CI of 1.01 to 1.67).

In another Taiwanese study [16], the CHA_2_DS_2_-VASc score (but not its individual components) was examined in about 6200 MHD patients with newly diagnosed AF. Unlike our study, they excluded patients with AF developed before they started MHD treatment. It was emphasized in this study that the predictive capacity of the CHA_2_DS_2_-VASc score in MHD patients for future strokes is eliminated due to their high mortality rate of other causes prior to the event of future strokes [16]. In our study, conversely, the predictability of future ischemic strokes by both CHA_2_DS_2_-VASc as a whole score and past ischemic events remained statistically significant even after adjusting for competing all-cause death risks. This discrepancy can be explained by the onset differences of AF and related clinical features (higher dialysis vintage, lower DM prevalence, lower comorbidity scores in their study compared to ours) as well as by the populations’ ethno-demographic differences (younger patients, more men in their study).

A history of prior ischemic strokes/TIAs raises the risk of future strokes in the CKD population, and we have shown that this association’s strength is highest in the MHD population (a more than eightfold increased risk for a subsequent stroke in our study). This is probably due to the following: first, the incidence of stroke is substantially higher among MHD patients than among earlier stage CKD patients [23]; second, the severity of risk factors for ischemic stroke unique to CKD patients, such as chronic inflammation and vascular calcification, is higher in MHD patients [24, 25]. Failure to take these risk factors into account, as well as assigning different weights of conventional risk factors in assembling an overall risk for ischemic stroke may explain why the CHA_2_DS_2_-VASc, the most commonly used stroke risk score in the general population, has limited validation in the MHD population. A literature review on stroke risk stratification by CHA_2_DS_2_-VASc in the MHD populations reported c-statistics of less than 0.70 in almost all studies [26] which are consistent with our results.

We found that the HAS-BLED score and its components did not predict bleeding risk discrimination in our study population. Two recent studies by Wang TK et al. [27] and Ocak G et al. [17] that tested HAS-BLED in dialysis patients have shown similar results. We additionally examined individual components of HAS-BLED as predictors. We did not find any advantage of the HAS-BLED components compared to the complete score in discriminating major bleeding risks. The possible reasons for HAS-BLED’s poor predictive ability in the MHD population are discussed in detail by OcakG et al. [17]. In our view, the main reason for this failure is that ESKD in itself increases the risk of bleeding. The risk of hemorrhage increases in a graded fashion with declining eGFR. The adjusted risk ratio of all-cause major hemorrhage in ESKD, with eGFR ≥90 ml/min per 1.73 m^2^ as a reference, is reported as 3.0 (95% CI of 1.3 to 6.6) to 5.5 (95% CI of 3.9 to 7.6) depending on the severity of albuminuria [28]. Therefore, discriminating low- versus high-risk patients by HAS-BLED score, as done in the general population, becomes impossible in MHD patients. It should be noted that even in the general population HAS-BLED is an auxiliary bleeding evaluation tool. A recent review of current guidelines for AF treatment reviewed updated guidelines among Europeans, Canadians, Asians, Americans, Koreans, New Zealanders and emphasized the agreement between them that there is no need to stop anticoagulants even if the HAS-BLED score is high, but to discuss with the patient the risks involved, correct the risk factors and conduct frequent surveillance [29].

A number of limitations inherent to observational research applies to our analysis. First, due to the observational approach, no definitive cause and effect relationship can be derived for any of the risk factors analyzed. Second, selection bias is typical in retrospective studies. A difference in the outcome incidence of interest between those who participated and those who did not would give biased results. This can affect generalizing our findings to the wider MHD population. Although the statistical power of the study to detect a difference of 1.0 points in the CHADS2-VASc score between groups with and without ischemic stroke was calculated as 90% (about 10% false acceptance of the null hypothesis) with an α error of 5%. A relatively low number of events prevented us from adjusting multivariable models to all potential confounders. However, the results obtained were very significant, the groups were quite homogeneous and further adjustments would not affect the results.

## Conclusions

In summary, ischemic stroke risks are high in MHD patients who had previous CVAs/TIAs. There is no need to calculate the CHA_2_DS_2_VASc score in these patients because it does not add value to the performance of prior CVAs/TIAs in predicting future strokes. Moreover, even high CHA_2_DS_2_VASc scores are less predictive in MHD patients without a history of CVAs/TIAs. Using CHA_2_DS_2_VASc scores in such patients when considering giving anticoagulants can expose them to unnecessary bleeding risks. HAS-BLED scores are not valid in MHD patients to predict major bleeding risks. Our results underline the need to seek new and unique approaches for MHD patients to balance the risks of thrombosis versus bleeding.

## Data Availability

The datasets used and/or analysed during the current study are available from the corresponding author (iliab@shamir.gov.il) on reasonable request.

## Abbreviations

MHD: Maintenance hemodialysis
ESKD: End stage kidney disease
CKD: Chronic kidney disease
CHF: Congestive heart failure
DM: Diabetes mellitus
AF: Atrial fibrillation
HR: Hazard ratio
CI: Confidence interval
CVA: Cerebrovascular accident
TIA: Transient ischemic attack
SD: Standard deviation
ROC: Receiver operating characteristic curve
ROC AUC: Area under the ROC curve
L +: Positive likelihood ratio
L-: Negative likelihood ratio
INR: International normalized ratio
